# 

**Published:** 1842-11

**Authors:** 


### CONTENTS

OF NO. V.

### ORIGINAL COMMUNICATIONS.

### ESSAYS AND CASES.

Art. I.—A Case of Compression of the Brain, reported to the Medical Society of Tennessee, at its 13th annual meeting in May, 1842. By John R. Wilson, M. D., of Davidson County. - - - - - 321

Art. II.—History of a fatal Case of Chronic Spinitis. By Calvin Smith, M. D., of Toledo, Ohio. - - 323

Art. III.—Observations on Scarlatina Anginosa, as it appeared at St. Clairsville, Ohio, in 1833. By Thomas Carroll, M. D., now of Cincinnati. - - 327

### REVIEWS.

Art. IV.—Animal Chemistry; or Organic Chemistry in its application to Physiology and Pathology. By Justus Liebig, M. D., Professor of Chemistry in the University of Giessen. Edited from the Author's MS., by William Gregory, M. D., Professor of Chemistry, King's College, Aberdeen. With additions, notes, and corrections, by John W. Webster, M. D., Erving Professor of Chemistry in Harvard University. 342

SELECTIONS FROM AMERICAN AND FOREIGN JOURNALS.

On the Incipient Stage of Cancerous Affections of the Womb. 380
Styptic Effect of Kreasote. - - - 383
The late Baron Larrey. - - - - 384
M. Louis on Typhoid Fever. - - - - 386
Ligature of the Primitive Iliac Artery. - - - 390
Devilliers and Chailley on Auscultation in Pregnancy. - 392
Post-Mortem Examination of a Soldier one hundred and two years old. - - - - - - 393
Lime Moxa. - - - - - - 394
Sciatica cured by Extract of Belladonna. - - 394
Removal of Discoloration of the Skin from Nitrate of Silver. 395
Employment of the Ergot of Rye in Cases of Paraphlegia. 395
The Employment of Strychnia in Amaurosis. - - 397

ORIGINAL INTELLIGENCE.

Kane's Elements of Chemistry. - - - - 398
Phrenology and Napoleon. - - - - 399

